# Physical biology of human brain development

**DOI:** 10.3389/fncel.2015.00257

**Published:** 2015-07-08

**Authors:** Silvia Budday, Paul Steinmann, Ellen Kuhl

**Affiliations:** ^1^Chair of Applied Mechanics, Department of Mechanical Engineering, University of Erlangen/NurembergErlangen, Germany; ^2^Department of Mechanical Engineering and Bioengineering, Stanford UniversityStanford, CA, USA

**Keywords:** neurodevelopment, connectivity, synaptogenesis, cortical folding, differential growth, lissencephaly, polymicrogyria

## Abstract

Neurodevelopment is a complex, dynamic process that involves a precisely orchestrated sequence of genetic, environmental, biochemical, and physical events. Developmental biology and genetics have shaped our understanding of the molecular and cellular mechanisms during neurodevelopment. Recent studies suggest that physical forces play a central role in translating these cellular mechanisms into the complex surface morphology of the human brain. However, the precise impact of neuronal differentiation, migration, and connection on the physical forces during cortical folding remains unknown. Here we review the cellular mechanisms of neurodevelopment with a view toward surface morphogenesis, pattern selection, and evolution of shape. We revisit cortical folding as the instability problem of constrained differential growth in a multi-layered system. To identify the contributing factors of differential growth, we map out the timeline of neurodevelopment in humans and highlight the cellular events associated with extreme radial and tangential expansion. We demonstrate how computational modeling of differential growth can bridge the scales–from phenomena on the cellular level toward form and function on the organ level–to make quantitative, personalized predictions. Physics-based models can quantify cortical stresses, identify critical folding conditions, rationalize pattern selection, and predict gyral wavelengths and gyrification indices. We illustrate that physical forces can explain cortical malformations as emergent properties of developmental disorders. Combining biology and physics holds promise to advance our understanding of human brain development and enable early diagnostics of cortical malformations with the ultimate goal to improve treatment of neurodevelopmental disorders including epilepsy, autism spectrum disorders, and schizophrenia.

## 1. Introduction

The average adult human brain has a volume of 1350 cm^3^, a total surface area of 1820 cm^2^, and an average cortical thickness of 2.7 mm (Pakkenberg and Gundersen, 1997). It contains approximately 100 billion neurons, of which 20 billion are located in the cerebral cortex (Herculano-Houzel, 2009). Each cortical neuron has on average 7000 synaptic connections to other neurons, resulting in a total of 0.15 quadrillion synapses and more than 150,000 km of myelinated nerve fibers (Pakkenberg et al., 2003). Gyrification, the folding of the cortical surface, is viewed as a mechanism to maximize the number of cortical neurons and minimize the total fiber length within the limited space inside our skull (Zilles et al., 2013).

In recent years the question what drives cortical folding has engaged researchers across various fields (Richman et al., 1975; VanEssen, 1997). After decades of biological research, physical forces are now increasingly recognized to play a central role in regulating pattern selection and surface morphogenesis (Smith, 2009; Bayly et al., 2013; Franze et al., 2013; Budday et al., 2014b; Ciarletta et al., 2014). While there is a general agreement on the importance of mechanical forces during neurodevelopment (Franze, 2014), to the present day, the physical biology of human brain development remains understudied and poorly understood (Bayly et al., 2014).

From a physical perspective, cortical folding is an instability problem of constrained differential growth in a multi-layered system (Goriely and BenAmar, 2005). From a biological perspective, three distinct phases contribute to differential growth: neuronal division and migration; neuronal connectivity; and synaptogenesis and synaptic pruning (Raybaud et al., 2013). The first phase of brain development spans throughout the first half of gestation and is characterized by the creation of new neurons–at rates of up to 250,000 neurons per minute–and their migration toward the outer brain surface (Blows, 2003). Not surprisingly, neuronal division and migration are associated with a noticeable cortical growth both in thickness and surface area (Sun and Hevner, 2014). However, until mid-gestation, the growth-induced cortical stress is too small to induce cortical folding and the cortical surface remains smooth (Budday et al., 2014b). The second phase spans from mid-gestation throughout 2 years postnatally and is dominated by the formation of neuronal connectivity. The new connections induce an excessive tangential expansion of the outer cortex (Huttenlocher and Dabholkar, 1997), the cortical stress increases, and the cortex begins to fold (Richman et al., 1975). At the same time, myelination reaches its peak and induces extreme white matter growth. The third phase spans throughout the entire lifetime and is associated with mild synaptogenesis, the formation of a few new connections, but mainly with synaptic pruning, the removal of unnecessary neuronal structures (Craik and Bialystok, 2006). Throughout this phase, the human cortex remains plastic, locally adapts its thickness, dynamically adjusts its stress state, and undergoes secondary and tertiary folding (Budday et al., 2015c).

In this review, we summarize biological mechanisms of neuronal division, migration, and connectivity with a view toward the physical phenomena of surface morphogenesis, pattern selection, and evolution of shape. We highlight how physical forces act as regulators in translating these cellular mechanisms into the gyrogenesis of the human brain. We demonstrate how computational modeling of differential growth can predict the classical pathologies of lissencephaly and polymicrogyria, and how the underlying model could be expanded toward other neurodevelopmental disorders including microcephaly and megalencephaly. We conclude with a critical discussion of the role of physics and biology in human brain development and identify future challenges and new frontiers.

## 2. Neuronal division and migration

Neurogenesis involves a precisely orchestrated sequence of cellular events. It begins with the formation of the neocortex at the rostral end of the neural tube, near the outer surface of the embryonic cerebral vesicle (Sidman and Rakic, 1973). In humans, the neural tube closes at embryonic day 30, during the fifth week of gestation (O'Rahilly and Müller, 2006). A primitive ventricular system begins to form and the amniotic fluid is trapped within the central canal. Neural tube closure initiates a rise in intraventricular fluid pressure, which marks the beginning of rapid brain enlargement.

**Figure 1:**
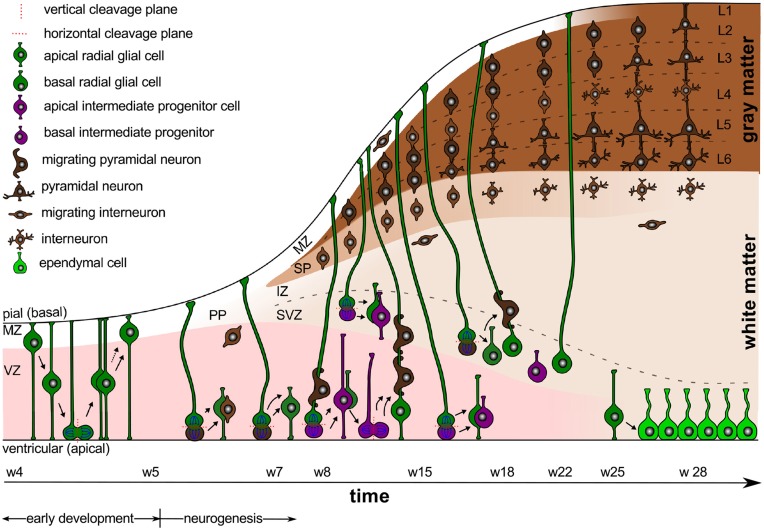
**Early development and neurogenesis**. Early development is characterized by interkinetic nuclear migration, an oscillatory process during which neuroepithelial cells divide symmetrically at the margin of the ventricle and undergo four phases. Early born neurons are referred to as interneurons and travel tangentially within the marginal and intermediate zones. Neurogenesis begins when progenitor cells switch from symmetric to asymmetric cell division. Apical progenitor cells in the ventricular zone and basal progenitor cells in the subventricular zone accumulate to become the major source of pyramidal neurons.

Intracranial pressure is now recognized as an important regulator of normal brain development (Desmond, 1985). In the absence pressure, the brain cavity volume enlarges less rapidly, brain tissue grows at a reduced rate, remains grossly disorganized, and tends to fold inward into the ventricular cavity (Desmond and Jacobson, 1977). In addition to the mechanical regulation through pressure, the cerebrospinal fluid provides biochemical regulation through diffusible extracellular signals, which modulate symmetric and asymmetric progenitor cell division during development and disease (Lehtinen et al., 2011). Figure 1 illustrates the timeline of early development and neurogenesis including cell division and cell migration.

### Early development is characterized through early proliferation

Between weeks 4 and 5, early development is characterized by interkinetic nuclear migration, an oscillation process during which neuroepithelial cells divide symmetrically at the margin of the ventricle and undergo four phases (Bystron et al., 2008): first, the cell nuclei position themselves at the basal, abventricular locations; then, they move toward the apical ventricular surface; next, they divide symmetrically into two new progenitor cells at the apical surface; and finally, they return toward their basal position. This early proliferation exponentially increases the number of progenitor cells, and ultimately results in both increased surface area and increased thickness of the ventricular zone.

Weeks 4–5 also mark the time period when the first primitive layer of meningeal cells becomes visible (O'Rahilly and Müller, 1986). There is increasing evidence that the fetal meninges play a critical role in brain development. While the mechanical role of the meninges is still not entirely understood, we now know that the dura mater, the arachnoid mater, and the pia mater are far more than protective layers around our brain: By releasing diffusible factors, the meninges form a major signaling center between the cortex and the skull to control the proliferation and migration of neural progenitors and neurons (Siegenthaler and Pleasure, 2011). Not surprisingly, disruption of meningeal function–either globally or locally–can result in complex brain pathologies including cobblestone malformations, lissencephaly, and polymicrogyria (Sun and Hevner, 2014).

### Gestational week 5 marks the onset of neurogenesis

Around gestational week 5, the progenitor cells in the ventricular zone, the radial glial cells, begin to switch from symmetric to asymmetric cell division (Iacopetti et al., 1999). Figure 1 indicates that symmetric cell division is associated with vertical cleavage planes, while asymmetric cell divisions is associated with horizontal cleavage planes (Haydar et al., 2003). During asymmetric division, one daughter cell remains in the ventricular zone as a radial glial cell, the other one becomes either a postmitotic neuron or an intermediate progenitor cell (Pontious et al., 2008). Intermediate progenitor cells eventually undergo terminal symmetric division to create pairs of postmitotic neurons (Noctor et al., 2004).

As Figure 1 suggests, we can classify radial glial cells and intermediate progenitor cells into two subpopulations, apical and basal: apical radial glial cells and apical intermediate progenitor cells form bipolar radial fibers between the apical and basal surfaces and reside in the ventricular zone (Sun and Hevner, 2014); basal radial glial cells and basal intermediate progenitor cells form a unipolar basal fiber and delaminate from the ventricular zone (Kowalczyk et al., 2009). Both types of basal cells are not attached to the ventricular surface and do not undergo interkinetic nuclear movement (Hansen et al., 2010). Accumulation of basal progenitor cells creates a distinct new compartment above the ventricular zone, the subventricular zone (Haubensak et al., 2004). Its asymmetrically dividing basal radial glial cells become an additional source of intermediate progenitor cells, which are especially important in species with larger brains and longer migration paths (Kriegstein et al., 2006). Asymmetrically dividing basal radial glial cells contribute significantly to cortical growth and folding (Lui et al., 2011).

The ventricular and subventricular zones form an active proliferate zone, the site of origin of pyramidal neurons. Pyramidal neurons eventually migrate outward along the radial glial cells to form the characteristic six-layered cortical structure from inside to outside (Raybaud et al., 2013). Newborn pyramidal neurons pause in the subventricular zone for up to 24 h before they migrate radially. This suggests that the subventricular zone synchronizes the migration of pyramidal neurons and interneurons (Lui et al., 2011).

Before the radial migration of pyramidal neurons begins, early-born cells of various type fill the space near the basal surface to form a dynamic, transient structure, the preplate (Bystron et al., 2008). Cells in the preplate are born before the first radially migrating pyramidal neurons and will either die or migrate tangentially to become inhibitory interneurons, either in the cortex or in the subcortex (DelRío et al., 2000). During early neurogenesis, asymmetric and symmetric cell division provoke a notable radial and tangential expansion of all proliferative zones.

### At gestational week 7, the cortical plate begins to develop

Around week 7, radially migrating neurons from the ventricular and subventricular zones initiate the development of the cortical plate. In its early stages, the cortical plate is divided into two layers, a thin superficial marginal zone and an underlying subplate. The marginal zone contains cells that have mostly migrated tangentially; those cells arrest the migration of radially migrating pyramidal neurons to guarantee the inside-out formation of the cortex (Rakic and Zecevic, 2003). The marginal zone will eventually form cortical layer 1 (Raybaud et al., 2013). The underlying subplate contains both, interneurons and postmigratory pyramidal neurons, which transiently connect with incoming axons until the cortex is ready to receive them (Molnár et al., 1998).

Cortical neurons accumulate above the subplate in an inside-out sequence: The earliest-born neurons are destined to become the innermost layer 6, the last-born neurons will become the outer layer 2. Early projection pyramidal neurons of the inner cortical layers originate from apical radial glial cells in the ventricular zone, whereas later neurons in the superficial layers progressively originate from secondary progenitor cells in the subventricular zone (Lui et al., 2011). The development of the whole cortical plate follows a temporal gradient of maturation across the hemisphere: first it forms in the most lateral part of the rostral telencephalic wall; approximately 1 week later, it develops in the dorsocaudal pole (O'Rahilly and Müller, 2006).

The cell-sparse compartment between the proliferative ventricular and subventricular zones and the cortical plate is known as the intermediate zone. It consists of radially and tangentially migrating cells, former preplate cells, and long-range axons (Bystron et al., 2008). The intermediate zone will eventually become white matter tissue. At this point, the proliferative zones still expand radially and tangentially, while the development of the cortical plate initiates a pronounced radial expansion of the cortex.

### Until gestational week 18, the cortex forms its six-layered structure

Between weeks 9 and 12, the human subplate thickens considerably and its cell density decreases (Carney et al., 2007). This phase is accompanied by a gradual thickening of the cortex. The morphology of each cortical neuron that has reached its final position reflects its developmental age (Bayer and Altman, 1991): young cortical neurons have an elongated cell body, a descending axon that connects with a neuron in the subplate, and an apical dendrite (Bystron et al., 2008); following the radial gradient, the age of the neurons increases, their cell bodies become progressively more rounded, and their dendrites elongate perpendicular to the cortical surface (Sidman and Rakic, 1973). This implies that older neurons in the deep cortical layers form connections earlier than younger neurons in the superficial layers.

Between weeks 13 and 15, the ventricular zone becomes progressively thinner as a large percentage of its cells moves outward (Sidman and Rakic, 1973). By this time, the neurons destined for the prospective middle layers of the cortex have arrived. Finally, at gestational week 18, we can clearly distinguish the radial organization of the neocortex with its six distinct layers. However, there is still a striking contrast between the bipolar shaped, immature, superficial cells and the complex shaped, mature, deeper neurons, which already possess elaborately branched dendrites (Sidman and Rakic, 1973). This might explain why in larger brains, which have a greater proportion of late-derived neurons, the outer cortical layers are disproportionately thickened (Hill and Walsh, 2005). At this point, no horizontal intracortical connections have developed yet (Noctor et al., 2001). The formation of the six-layered cortex mainly involves the radial expansion of the cortical plate and subplate, while the proliferative zone begins to attenuate.

### After gestational week 22, the cortical plate differentiates

The period after week 22 is the most significant time for areal, laminar, and cytological differentiation of the cortical plate. Gyral formation begins around week 24 at the parieto-occipital and central sulci (Takahashi et al., 2012). By week 25–27, the ventricular zone has reduced to a one-cell-thick ependymal layer. The subventricular zone, which is still proliferating, will now become the major source of cortical neurons (Zecevic et al., 2005). The subplate has reached its maximum thickness and begins to attenuate (Kostović et al., 2002); yet, some residual subplate neurons persist throughout life as interstitial neurons in the white matter tissue (Bystron et al., 2008).

By week 28, layer 1 has fully developed. It contains a few neurons and is largely filled with arborizations of apical dendrites and intrinsic tangential axons. The cells that had initially arrested the migration of pyramidal neurons during neuronal migration have now disappeared (Raybaud et al., 2013). Once neuronal migration is complete, the radial glial cells in the subcortical layers disappear or become astrocytes (Misson et al., 1991). Interneurons keep migrating until the last trimester when the migration of pyramidal neurons ceases (Raybaud et al., 2013). They travel tangentially within the marginal and intermediate zones and can enter the cortical plate from either location to form local circuits with cortical pyramidal neurons. Weeks 24–34 mark a critical period for axonal elongation and maturation in the developing white matter (Holland et al., 2015). This period is associated with the beginning of myelination, which transforms the original intermediate zone into white matter tissue. During differentiation, neuronal dendrites elongate perpendicularly and cause a significant tangential expansion of the cortical plate. At the same time, the subplate attenuates and the intermediate zone transforms into white matter tissue. The resulting differential growth between the cortical plate and inner layers of the brain results in an increase in cortical stress and, eventually, in the onset of cortical folding.

Taken together, numerous interacting modes of cell migration shape the developing neocortex. A proper radial and tangential development of the mammalian brain requires different types of progenitor cells, depending on species and stage of development. As a common paradigm, radial glial cells tend to expand the cortex tangentially, whereas intermediate progenitor cells tend to fill the cortical layers radially. In addition to the radial gradient of development, there is a pronounced tangential gradient from primary, via secondary, to highly associative cortices. This tangential gradient results in a similar developmental gradient of connectivity and gyrification, and, after term, in a similar gradient of cortical myelination.

## 3. Neuronal connectivity

**Figure 2:**
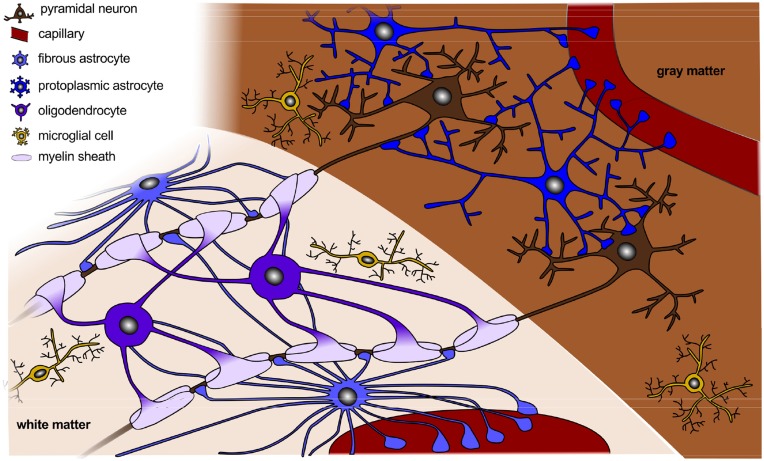
**Organization of a fully developed brain**. In white matter, myelinated axons allow for rapid nerve impulse conduction; intermediate oligodendrocytes connect and form several myelin sheaths. Fibrous astrocytes ensure supply of nutrients and synaptic processing. In gray matter, neurons form synapses with each other and with protoplasmic astrocytes. In both white and gray matter, microglial cells contribute to clearance of debris and synapse remodeling.

Figure 2 sketches the final organization of the brain, once all components have fully developed and connected. Besides cell division and cell migration, the formation of connections is a major factor of normal brain development. Before midgestation, there is essentially no direct connection between the cortex and the rest of the central nervous system. At this point, there are only indirect connections between cortical and subcortical structures and the subplate (Haynes et al., 2005). As connectivity develops, axons form and multiply their branches. During the second half of gestation, axons extend branches to numerous cortical and subcortical targets, until each neuron connects with thousands of other neurons (Raybaud et al., 2013). The mechanical forces during this phase of axonal elongation have been extensively studied (Suter and Miller, 2011; O'Toole et al., 2015). They have lead to the popular but controversial hypothesis of surface morphogenesis through axonal tension and compact wiring (Mitchison, 1991). Axonal tension, a mechanisms to bring functionally related units topographically closer together (VanEssen, 1997), can explain folding with realistic stiffness ratios, but disagrees with dissection experiments (Xu et al., 2010). Differential growth, a mechanism to release residual stresses by surface buckling (Ronan et al., 2014), agrees with dissection experiments, but requires unrealistic stiffness ratios (Richman et al., 1975). Combining both mechanisms has motivated theories of stretch- and stress-driven growth, concepts that agree with both realistic stiffness ratios and maximum principal stress distributions (Bayly et al., 2013; Budday et al., 2014b).

The most prominent connective structure in the human brain is the corpus callosum, a wide, flat bundle of more than 200 million contralateral axons, which connect the left and right cerebral hemispheres (Luders et al., 2010). The corpus callosum begins to differentiate as a commissural plate around week 8. In the human embryo, the earliest callosal axons appear around week 12, and the adult callosal morphology is achieved around week 20 (Achiron and Achiron, 2001). Agenesis of the corpus callosum, a rare but severe congenital disorder in which the corpus callosum is partially or completely absent, is often associated with profound intellectual disabilities (Palmer and Mowat, 2014).

In the cortex, the formation of connections follows the radial gradient of the inside-out proliferation: First, the early-born deep layer 6 neurons extent branches to the basal gray matter; around week 17, layer 5 neurons form connections with the internal capsule, the brainstem, and the spinal cord; between weeks 22 and 27, layer 4 neurons receive thalamo-cortical connections; last, from week 28 to 32, layer 3 and 2 neurons form intrahemispheric, cortico-cortical association fibers and interhemispheric, commissural connections (Raybaud et al., 2013). Subplate neurons participate in both local and long-distance axonal connections. From week 32 to 47, the development of short horizontal connections in the cortical gray matter and in the subcortical white matter results in a horizontal rather than radial final layering pattern of the neocortex (Marin-Padilla, 1970). The deep layers 6 and 5 project single axons, whereas the superficial layers 4, 3, and 2 and the subplate receive multiple incoming fibers. This implies that connectivity-driven tangential growth mainly affects the superficial layers and the subcortical white matter tissue (Raybaud et al., 2013).

Not only neurons, but also other cellular components such as astrocytes, oligodendrocytes, microglial cells, and capillaries play a crucial role in the proper formation of neuronal connectivity (Barres, 2008). Actually, radial glial cells in the subplate and in the intermediate zone not only produce neurons but also astrocytes and oligodendrocytes (Mizutani et al., 2007). The creation and growth of these additional cells induces an additional volume expansion in the corresponding layers. In the following, we outline the individual development of each cell type.

### Astrocytes guide the migration of developing axons and neurons

Astrocytes are a sub-type of glial cells in the central nervous system. They play a major role in synaptic transmission and information processing, they guide the migration of developing axons and neurons, and have extensive contacts with blood vessels (Powell and Geller, 1999). Astrocytes develop during the second half of gestation after the mass production of neurons (Freeman, 2010), when the radial glial cells switch from generating exclusively neurons to generating astrocytes (Morrow et al., 2001). The timing of the neuron-astrocyte switch indirectly affects the number of astrocytes in the adult brain (Molofsky et al., 2012). The generation and expansion of astrocytes is largely completed by early postnatal stages, but astrocytes continue to elaborate and refine their branches after birth during the active period of synaptogenesis (Lund and Lund, 1972). Eventually, astrocytes become the most abundant cell type in the human brain (Freeman, 2010).

Morphologically, we can distinguish two types of astrocytes as illustrated in Figure 2, protoplasmic and fibrous: protoplasmic astrocytes are primarily located in gray matter tissue and exhibit fine irregular branches in a globoid distribution; fibrous astrocytes are widely spread throughout the white matter tissue and exhibit numerous regular cylindrical fibers (Vaughn and Peters, 1967). Each astrocyte occupies a unique spatial domain and forms discrete borders with its neighbors (Bushong et al., 2002). Human astrocytes are larger, structurally more complex, and more diverse than astrocytes in rodents. The ratio of astrocytes to neurons significantly varies between species: It is 1:6 in worms, 1:3 in rodents, and 1.4:1 in humans (Nedergaard et al., 2003). This suggests that the astrocyte-to-neuron ratio increases with cognitive skills.

### Oligodendrocytes support neural migration and myelinate axons

Oligodendrocytes are a type of neuroglial cells. They provide structural support and form a myelin sheath around the axon to enable rapid impulse propagation (Freeman, 2010). The first oligodendrocyte progenitor cells form in the ventricular and subventricular zones around week 10, and their formation reaches a peak around week 15 (Jakovcevski et al., 2009). Oligodendrocyte progenitor cells first differentiate into pre-myelinating oligodendrocytes and then transform into mature myelin-forming cells (Pfeiffer et al., 1993). Pre-oligodendrocytes develop in the subplate, in the intermediate zone, and in the cortex by the end of the second trimester. After week 30, mature oligodendrocytes sparsely appear in the intermediate zone, before they increase diffusely after week 40. In humans, significant myelination only occurs after birth supported by a last wave of oligodendrocyte progenitor cells in the postnatal cortex (Kessaris et al., 2006). All oligodendrocytes travel long distances before they arrive at their final destination (Bradl and Lassmann, 2010). Although the vast majority of oligodendrocyte progenitor cells differentiates to myelin-producing oligodendrocytes, some inactive oligodendrocyte progenitor cells persist into late adulthood.

Myelination occurs early during oligodendrocytic differentiation within a narrow window of 12–18 h (Barres, 2008). It is regulated by the electrical activity of neurons: Increasing neuronal firing and axonal signaling enhances myelination (Diemel et al., 1998). Oligodendrocytes selectively wrap axons with diameters larger than 0.2 μm (Simons and Trajkovic, 2006). At the peak of myelination, oligodendrocytes can create up to three times their weight in membrane per day (McLaurin and Yong, 1995).

Oligodendrocytes and their myelin sheaths are more susceptible to damage than any other cell type in our brain. After demyelination caused by disease, for instance in multiple sclerosis, axons can retrieve new myelin sheaths. During remyelination, inactive adult oligodendrocyte progenitor cells proliferate, switch from their quiescent state to a regenerative phenotype, and generate new oligodendrocytes (Franklin and Ffrench-Constant, 2008). The process of remyelination is similar to myelination during development, except for longer cell cycle times, slower migration rates, and thinner and shorter sheath segments (Ludwin and Maitland, 1984). This suggests that axons with unusually thin myelin sheaths are a pathological hallmark of remyelination.

In rodents, myelination begins already during neurogenesis. In humans, it mainly occurs after birth, when neuronal migration has ceased and primary and partially secondary folds have formed. Myelination in rodents only takes a few weeks, compared to decades in the humans (Yakovlev and Lecours, 1967). Although the absolute number of oligodendrocytes in humans is significantly larger than in rodents, the oligodendrocyte density per white matter volume in humans and rodents is remarkably similar (Bradl and Lassmann, 2010).

### Microglial cells control neuronal proliferation and differentiation

Microglial cells contribute to neuronal proliferation and differentiation, clear debris, and remodel synapses (Harry, 2013). Unlike all other cell types in our brain, microglial cells are not differentiated from neural stem cells (Yang et al., 2013); they derive from myeloid progenitor cells migrating through the blood vessels (Harry, 2013). By migrating along radial glial cells, white matter tracts, and the vasculature (Rezaie and Male, 1999), microglial cells reach the ventricular zone by week 6, extent into the marginal zone by week 8, and spread into the subventricular zone, the intermediate zone, and the subplate around week 16 (Raybaud et al., 2013). As neurons become established in the brain, the need to clear excess neurons is reduced, and microglial cells undergo a morphological transition from amoeboid to heavily branched (Monier et al., 2006). By week 35, the cells display a fully developed, ramified morphology (Esiri et al., 1991). Microglial cells located in gray matter have a small, dense nucleus with fine branches extending in multiple directions; those in white matter have an elongated cell nucleus and align their fibers with neighboring axons. The majority of microglial cells, about 95%, appears within the first 2 weeks post-natally (Alliot et al., 1999). Eventually, microglial cells comprise approximately 15–20% of the total number of brain cells (Carson et al., 2006). In general, the population of microglial cells correlates with the presence of apoptotic cells and blood vessels (Rezaie and Male, 1999).

### Vasculogenesis ensures supply with oxygen and nutrients

The vasculature of the brain ensures its supply with oxygen and nutrients. The cerebral vesicles begin to form around week 5. During week 6–7, blood channels develop to form a dense capillary bed within the ventricular zone. Since the development of the vascular system closely correlates with cell proliferation and connectivity, it follows the radial gradient from inside to outside and the tangential gradient from primary to secondary and highly associative cortices (Norman and O'Kusky, 1986). During the first half of gestation, the capillary bed is limited to the ventricular and subventricular zones (Allsopp and Gamble, 1979). By week 15, first capillaries appear in the intermediate zone, the subplate, and the deep cortical layers (Marín-Padilla, 2012). From week 22 to 27, the cortical capillary bed progresses from the deep layers toward the surface. Its density increases continuously until week 47. With the attenuation of the proliferate ventricular and subventricular zones, their capillary beds regress (Allsopp and Gamble, 1979). In the white matter, the microvascular density remains low until term (Ballabh et al., 2004).

### Synaptogenesis and synaptic pruning shape the cerebral cortex

Synaptogenesis, the formation of synapses between the individual neurons, begins before week 27, but occurs mainly after birth, concurrent with the growth of dendrites and axons and with the myelination of axons in the subcortical white matter (Huttenlocher and Dabholkar, 1997). Neurons are not born with the ability to receive synapses, but acquire this ability for instance through contact with astrocytes (Clarke and Barres, 2013). Synaptogenesis consists of three phases: first, immature synapses form between axons and dendrites; then, synapses undergo maturation and convert from a silent to an active state; finally, the synaptic number is reduced to refine the neuronal connections within the circuit (Craige et al., 2006). In humans, synaptic pruning, the process of synapse elimination, starts near the time of birth and is completed by the time of sexual maturation. It is influenced by environmental factors and is widely thought to represent learning (Craik and Bialystok, 2006).

Postnatally, from infancy to adulthood, our brain increases in size by a factor five (Craik and Bialystok, 2006). Throughout this period, the total number of neurons remains virtually unchanged. Postnatal growth is mediated almost exclusively by the myelination of axons and by the growth of synaptic connections. Humans form more synapses than rodents, which contributes to the greater capacity of the human brain to learn and adapt (Preuss et al., 2004).

## 4. Gyrogenesis

**Figure 3:**
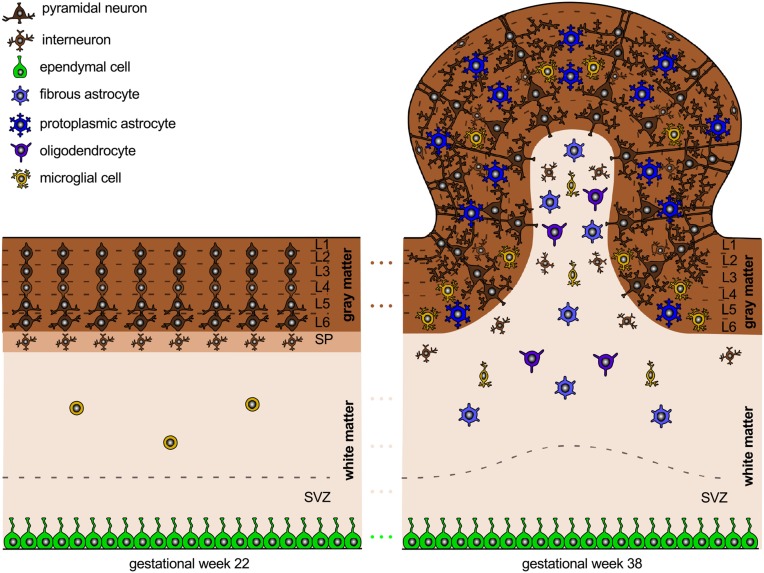
**Neural connectivity and gyrification**. The connection process follows the radial gradient of the inside-out proliferation. It begins in layer 6 and propagates outward toward layer 2. Connectivity-driven tangential growth mainly affects the superficial layers and triggers the formation of gyri and sulci. The subventricular zone is thicker at sites of growing gyri and thinner beneath developing sulci; both the subplate and the cortex are thicker at the crown of gyri than at the bottom of sulci.

While the early developmental processes are relatively well-characterized on the cellular level, later development of the entire brain with its distinct folded surface morphology remains less well-understood (Ronan et al., 2014). Figure 3 illustrates the structural changes that take place in our brain after its six cortical layers have formed.

### Gyrification begins around mid-gestation

Around week 23, the first primary sulci begin to form. Primary gyrification follows the same tangential gradient as proliferation and connectivity, from primary via secondary to high-association cortices. Through term, secondary sulci extend concentrically around the primary sulci. After term, tertiary sulci develop together with short association fibers. Gyrification typically begins slightly earlier in the right hemisphere than in the left (Raybaud et al., 2013).

The expansion of the cortex is an important regulator of brain folding. Several cellular processes correlate with gyral development: cell division in the subventricular zone is most abundant in highly folded regions (Lukaszewicz et al., 2005); the subventricular zone, which hosts basal radial glial cells and intermediate progenitor cells that will eventually differentiate into brain cells, is thicker at sites of growing gyri and thinner beneath developing sulci; both the subplate and the cortex are thicker in the crowns of gyri than underneath sulci (Kostović et al., 2002). Besides cell division, cell survival and cell death can significantly affect brain size and brain morphology. During normal brain development, apoptosis, the programmed death of neurons, cumulatively occurs at sites of developing sulci, while the late migration of superficial neurons is more prominent at the crowns of gyri (Smart and McSherry, 1986). Increased cell death in the exponentially growing early progenitor population can severely reduce the number of neurons and, vice versa, decreased death of progenitor cells exponentially increases the number of neurons. The latter phenomenon can even induce convoluted cortices in otherwise lissencephalic brains (Haydar et al., 1999).

In relation to the timelines of cell division and migration in Section 2 and connectivity in Section 3, gyrification occurs simultaneously with the late migration of superficial neurons and with the formation of neural connectivity as the number of astrocytes, oligodendrocytes, and microglial cells increases.

### Cortical folding is a mechanical instability driven by differential growth

A physics-based approach, which has recently gained increased attention, attributes cortical folding to differential growth between the cortex and the subjacent intermediate zone that later transforms into white matter tissue (Richman et al., 1975). The theory assumes that the early radial expansion of the cortical plate is relatively homogeneous across the thickness and does not evoke cortical folding. The later tangential expansion through the formation of horizontal, intracortical connections, however, is limited to the superficial cortical layers 1 through 4, and constrained by the inner layers 5 and 6 and by the underlying white matter. This creates compressive stresses, which may induce surface buckling to release the compression in the superficial layers (Biot, 1937).

The significance of the connectivity-driven tangential expansion of the superficial layers is supported by the fact that by week 18, when the cortex is still smooth, only radial but no tangential intracortical connections have formed (Noctor et al., 2001). Physical models of differential growth–with a morphogenetically growing outer surface on a stretch-driven growing inner core–can provide a mechanistic understanding of various features of brain folding (Bayly et al., 2014; Budday et al., 2014b). These models use the continuum theory of finite growth based on the concept of fictitious configurations (Lee, 1969). They introduce a second order tensor, the growth tensor, as an internal variable to record the history of the growth process (Rodriguez et al., 1994). Within this theory, the overall deformation gradient becomes the product of the elastic tensor, which reflects the reversible mechanical deformation, and the growth tensor, which reflects the biological process of growth (Ambrosi et al., 2011). Key to this theory is the fact that only the elastic part of the deformation generates stress. Once the stress in the cortical layer has reached a critical value, it is energetically more efficient for the cortex to bend rather than to be compressed (Cai et al., 2011). At this critical instability point, around week 23 in the healthy human brain, the cortex begins to fold (Moulton and Goriely, 2011). We can predict this folding analytically using simplified bi-layered models (Cao and Hutchinson, 2012), or computationally using elliptical or ellipsoidal models within a finite element analysis. While analytical models provide valuable insight into the critical conditions at the onset of folding, they fail to predict the interaction of gyri and sulci beyond the instability point (Balbi et al., 2015). Computational modeling can predict advanced surface morphologies and ultimately help correlate neurodevelopmental disruptions on the cellular level with structural malformations on the organ level (Budday et al., 2014a).

Although cortical folding does not occur before neuronal migration has ceased and all cortical layers have been established, cellular processes during neurogenesis play a major role in pattern selection (Raybaud et al., 2013). Increased asymmetric cell division in early brain development, for example, results in a reduced number of progenitor cells and neurons; fewer neurons migrate toward the cortical plate and will later expand tangentially (Sun and Hevner, 2014). A reduced number of progenitor cells not only reduces the number of migrating neurons, but may also reduce the number of radial migration paths; fewer neurons distribute over fewer radial glial cell fibers. This suggests that altered progenitor proliferation affects both the radial and the tangential organization of the cortical plate.

### Increasing the cortical thickness increases the gyral wavelength

**Figure 4:**
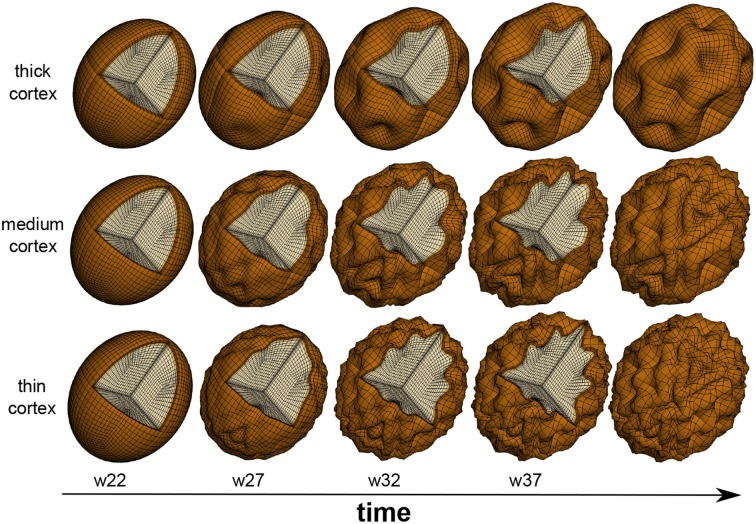
**Physics-based modeling of differential growth**. The surface morphology varies significantly with cortical thickness and gestational time. Decreasing the cortical thickness, from top to bottom, increases the number of folds and decreases the gyral wavelength. After the onset of folding around mid-gestation, the formation of new connections causes an excessive tangential expansion of the cortex, which increases the folding amplitude and surface complexity, from left to right.

Physics-based models predict that the gyral wavelength increases with increasing cortical thickness (Allen, 1969; Bayly et al., 2013), which is mostly determined by the radial organization of the cortical plate at the onset of folding. These models were initially developed to explain geological folding (Biot, 1937) and have recently been adopted for cortical folding (Bayly et al., 2014; Budday et al., 2014b). Figure 4 illustrates how changes in cortical thickness can modulate surface morphogenesis: the thinner the cortex, the shorter the gyral wavelength (Goriely et al., 2015). Growth-induced instabilities are initiated at the mechanically weakest spot (BenAmar and Goriely, 2005; Goriely and BenAmar, 2005). During neurogenesis, regional differences in the thickness of the subventricular zone could result in a heterogeneous cortical thickness, and folding would first occur in regions where the cortex is thinnest (Le Gros Clark, 1945). During early neuronal migration, thickness variations of the subventricular zone could serve as early markers of the developing folding pattern (Ciarletta et al., 2014). Taken together, it seems likely that small regional thickness variations in the newly established six-layered cortex largely shape primary folding. Other factors including local differences in synaptogenesis and synaptic pruning, and changes in mechanical properties due to myelination, rather gain significance during the later secondary and tertiary phases of cortical folding (Budday et al., 2015c).

**Figure 5:**
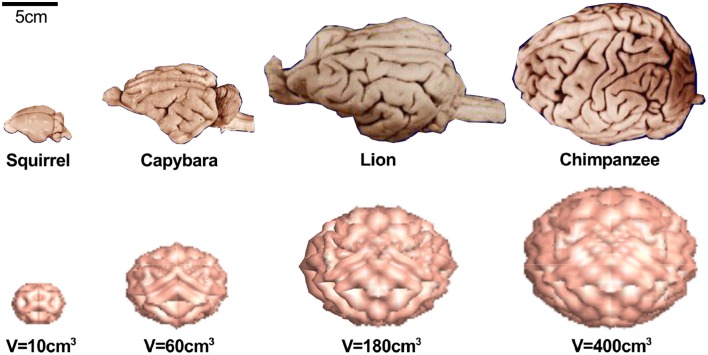
**The mammalian brain varies significantly in size, shape, and convolutional complexity, but only marginally in cortical thickness (Welker et al., 2015) (top)**. Physical models and computational simulations of growing brains with varying volume and constant thickness predict that the surface complexity increases with absolute size (Budday et al., submitted) (bottom). Our model predicts 22 major folds for the squirrel brain, 48 major folds for the capybara brain, 76 major folds for the lion brain, and 92-folds for the chimpanzee brain.

Figure 5 demonstrates that not only the cortical thickness itself, but also the ratio of cortical thickness to total brain volume plays an essential role in pattern selection (Le Gros Clark, 1990). The mammalian brain varies significantly in size, shape, and convolutional complexity, but only marginally in cortical thickness (Welker et al., 2015). Physical models and computational simulations of growing brains with varying volume and constant thickness predict that the surface complexity increases with absolute brain size (Budday et al., submitted). A comparative simulation of squirrel, capybara, lion, and chimpanzee brains with volumes on the order of 10, 60, 180, and 400 cm^3^ predicts the emergence of 22, 48, 76, and 92 major folds. If both total volume and cortical thickness were to increase proportionally, brains morphology would scale isometrically and similar folding patterns would emerge (Zilles et al., 2013). In agreement with geometric scaling studies of brain morphology, the computational model explains why increased brain volume at constant cortical thickness enhances folding (Hofman, 1989). The simulations are consistent with the observation that an increased thickness of the subventricular zone and an increased proliferation of progenitor cells increase the cortical thickness, cortical surface area, and brain size, but do not affect cortical folding (Sun and Hevner, 2014). In addition to the organization of the cortical layers at the onset of folding, the connectivity of the individual layers plays a crucial role throughout the folding process: radially forming connections primarily increase the cortical thickness, whereas horizontally forming connections induce tangential expansion, which will eventually enhance cortical folding (Moulton and Goriely, 2011).

### Increasing the cortical stiffness increases the gyral wavelength

Physics-based models predict that the gyral wavelength increases with the third root of the stiffness contrast between cortex and subcortex (Bayly et al., 2013). From an analytical point of view, growth-induced surface buckling requires that the stiffness of the gray matter layer is equal or larger than the stiffness of the white matter core (Cao and Hutchinson, 2011; Budday et al., 2015a). Motivated by these analytical considerations, the simulations in Figures 4, 5 use a stiffness contrast of three (Budday et al., 2014a). Throughout the past decade, numerous series of mechanical tests have been performed to characterize the stiffness contrast between gray and white matter tissue (Bilston, 2011; Miller, 2011). Testing brain remains challenging because of its extreme softness, its remarkable time dependence, and its microstructural architecture. On the small scale, scanning force microscopy revealed that gray matter was about twice as stiff as white matter, on the order of 100 vs. 50 Pa for ultra thin mouse spinal cord slices (Koser et al., 2015) and 500 vs. 250 Pa for rat cerebellum slices (Christ et al., 2010). On a larger scale, mechanical indentation tests found the opposite with gray matter about one third softer than gray matter, 1.8 vs. 1.2 kPa (Kaster et al., 2011) and 2 vs. 3 kPa (van Dommelen et al., 2010) for porcine brain and 1.9 vs. 1.4 kPa for bovine brain (Budday et al., 2015b). Discrepancies in these measurements not only reflect the extreme strain rate sensitivity of brain tissue, but also its non-linear behavior and its compression stiffening (Mihai et al., submitted). *In vivo*, magnetic resonance elastography suggests that mature gray and white matter are rather indistinguishable with shear stiffnesses on the order of 3 kPa in ferrets (Feng et al., 2013), and 3.1 vs. 2.7 kPa in humans (Green et al., 2008).

The microstructure of brain tissue–and with it the mechanical properties–change drastically during early brain development: While gray matter remodels from a radial to a tangential organization and intercellular cross-linking increases significantly during the third trimester, the degree of cross-linking in white matter remains low until term (Raybaud et al., 2013). This suggests that, during gyrification, between weeks 22 and 38, white matter is softer than gray matter, but then stiffens after term when myelination and the formation of astrocytic branches give rise to a highly cross-linked microstructure. White matter stiffening during brain development could not only explain the observed discrepancies in stiffness contrast (Budday et al., 2015b; Koser et al., 2015), but also the folding at low stiffness contrasts in agreement with the Biot condition (Biot, 1963).

The realistic prediction of cortical folding with the help of physics-based models requires the clarification of the mechanical properties of gray and white matter tissue–not only in the mature, but more importantly in the developing brain. Ultimately, this could unravel an important point of criticism against the hypothesis of differential growth, the fact that instabilities would manifest themselves in the form of creases rather than folds if gray matter was softer than white matter at the onset of the instability (Hutchinson, 2013). Since myelination already starts during neurogenesis in rodents (Rockland and DeFelipe, 2011), an increase in white matter stiffness with increased myelination could also explain why rodent brains are less folded than mammalian brains.

## 5. Malformations of cortical development

Malformations–as a result of interrupted cortical development–are common causes of mental disorders including developmental delay and epilepsy (Barkovich et al., 2012). However, the clinical features are quite ambiguous: similar structural abnormalities can cause diverse symptoms. For example, the same brain regions are affected by gray matter loss in patients with different psychiatric conditions such as schizophrenia, major depression, and addiction (Goodkind et al., 2015). The severity of cognitive abnormalities scales with the degree of cortical dysplasia. This suggests that brain function is closely correlated to brain structure and that it is essential to understand how and why structural abnormalities form. In addition to the pathologies that originate from disrupted neurodevelopment, several diseases are caused by dysfunction of mature brain cells. A typical example is oligodendrocyte loss associated with demyelination in multiple sclerosis. In the following, we focus on pathologies associated with neurodevelopment and illuminate the underlying mechanisms of four malformations, which originate during the early stages of neurogenesis.

### Lissencephaly is a migration disorder associated with a smooth brain

Lissencephaly literally means smooth brain and is associated with abnormal neuronal migration (Barkovich et al., 2005). As a result, migrating neurons fail to split the preplate into marginal zone and subplate; instead they form layers below the preplate with a reverse outside-in pattern (Sheppard and Pearlman, 1997). Large numbers of neurons do not even reach the cortical plate and diffusely deposit anywhere between the ventricular and pial surfaces (Friede, 1989); others overmigrate in the marginal zone and in the meninges. This implies that cortical lamination is completely disrupted.

The cortex of the lissencephalic human brain is significantly thickened–10 to 20 mm compared to 2–4 mm in normal brains–and typically consists of only four layers: an outer marginal layer 1, a superficial cellular layer 2 with numerous large and disorganized pyramidal neurons, a variable cell sparse layer 3, and a thick deep cellular layer 4 composed of medium and small neurons (Golden and Harding, 2004). The critical time window for the initiation of lissencephalic aberrations precedes gyrification, between weeks 11 and 13 (Aronica et al., 2012). Macroscopically, the classical hallmark of the disease becomes apparent only after week 22, when cortical folds fail to form: The connectivity-driven expansion is distributed over a significantly thicker cortex, which reduces the amount of compressive stresses and suppresses cortical folding. This suggests that the disease originates from disruptions during early brain development and neurogenesis, not from disruptions of the folding process itself.

**Figure 6:**
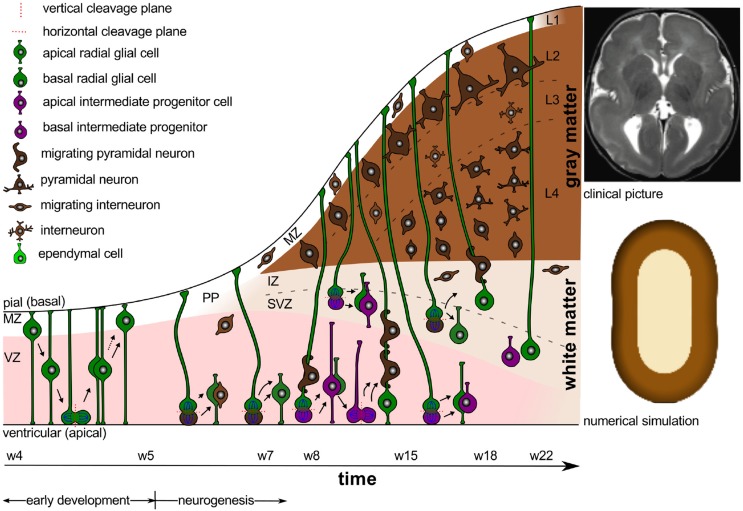
**Lissencephaly**. Cortical malformations result from abnormal neuronal migration during the early stages of neurogenesis. The extensively thickened cortex typically only consists of four disorganized layers. Consistent with the clinical picture of lissencephaly (Budday et al., 2014a) (top right), the numerical simulation (Budday et al., 2014b) (bottom right) predicts the absence of folds for extensively thickened cortices.

Figure 6 illustrates the abnormal neuronal migration during lissencephaly. Instead of forming six organized cortical layers as illustrated in Figure 1, the lissencephalic brain typically only forms four disorganized, thickened layers. Consistent with the pathology of lissencephaly (Budday et al., 2014a), the computational simulation of differential growth (Budday et al., 2014b) predicts that a considerably thickened cortex fails to fold since its growth-induced compressive stresses are too small to induce buckling.

### Polymicrogyria is an organization disorder associated with many small folds

Polymicrogyria is a developmental malformation of the human brain characterized by an excessive number of small folds. It evolves from abnormal development or loss of neurons in the deep cortical layers after completion of neuronal migration (Friede, 1989). Typically, layer 5 is damaged and the superficial layers overfold and fuse during gyrogenesis (Judkins et al., 2011). Deep layer neurons might retain a radial distribution without any laminar organization (Ferrer, 1984). This suggests that connectivity-driven growth after week 22 is mainly limited to the superficial layers, whereas the deep layers do not grow at all. The thinning of the cortex is magnified by the fact that the damaged deep cortical layers impede perfusion between week 20 and 24 and prohibit migration of astrocytes and oligodendrocytes (Ferrer and Catalá, 1991). This causes an undersupply of the superficial layers and cumulative cell death. Ultimately, polymicrogyric brains exhibit very thin cortices with an excessive number of small irregular folds (Guerrini et al., 2008). We can distinguish between layered polymicrogyria and unlayered polymicrogyria.

**Figure 7:**
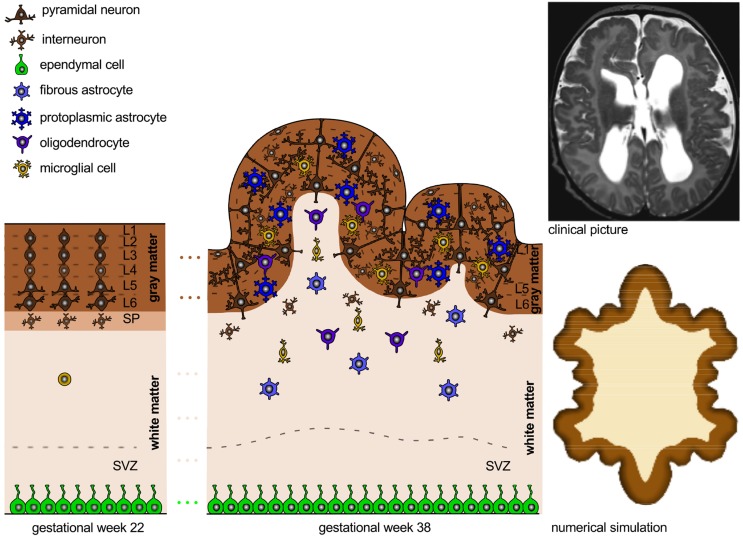
**Polymicrogyria**. Cortical malformations resulting from abnormal neuronal organization. Increased cellular necrosis and disturbed neuronal connectivity result in a thinned cortex with an excessive number of small irregular folds. Consistent with the clinical picture of polymicrogyria (Budday et al., 2014a) (top right), the numerical simulation (Budday et al., 2014b) (bottom right) predicts the emergence of many, small irregular folds for thin cortices and reduced growth of inner layers.

Figure 7 illustrates a layered polymicrogyric cortex. In comparison to the healthy human cortex in Figure 3, the polymicrogyric cortex consists of only four layers: an outer molecular layer corresponding to the healthy layer 1, a second cellular layer resulting from the fusion of the healthy layers 2 through 4, a third layer devoid of neurons representing the damaged layer 5, and a deep cellular layer similar to the healthy layer 6 (Gilbert-Barness and Debich-Spicer, 2008). Consistent with the pathology of polymicrogyria (Budday et al., 2014a), the computational simulation of differential growth (Budday et al., 2014b) predicts the emergence of small superficial folds as a result of cortical thinning and reduced growth in the subjacent layers.

### Microcephaly is a neurodevelopmental disorder associated with a small brain

Microcephaly is a rare developmental disorder associated with an abnormally small brain. In microcephalic brains, an altered cleavage plane during progenitor cell division reduces vertical, symmetric cell division and promotes horizontal, asymmetric cell division. This reduces the overall number of progenitor cells and neurons and decreases the cortical volume. In mild cases, the decrease in the total number of neurons is not accompanied by a commensurate loss of cortical folding (Bond et al., 2002). In more severe cases, however, an additional increase of apoptosis results in thinned cortices and abnormally small convolutions (Barkovich et al., 2005). These observations are consistent with the computational model of differential growth, which predicts that a thinner cortex in a proportionally smaller brain does not affect the folding pattern, whereas a markedly thin cortex in a normal brain evokes abnormally small convolutions.

### Megalencephaly is a neurodevelopmental disorder associated with an enlarged brain

Megalencephaly is a rare developmental disorder associated with an abnormally large brain. In megalencephalic brains, during early brain development, shortened cell cycles and increased cell cycle reentry increase the number of progenitor cells and neurons (Sun and Hevner, 2014). In mild cases, megalencephalic brains are enlarged but otherwise normal. In severe cases, the larger brain size is accompanied by enhanced folding, potentially brought about by decreased apoptosis (Barkovich et al., 2005). The increased number of progenitor cells and neurons leads to a proportional increase of brain volume and cortical thickness, which evokes normal folding patterns corresponding to the physical model of differential growth. Decreased apoptosis could initiate a faster tangential expansion of the cortex, which we could model by increasing the cortical growth rate. This would mimic neurons that normally undergo apoptosis, but now still form cortico-cortical connections. Consistent with the pathology of severe megalencephaly, an increase of the growth ratio between the superficial layers, which form horizontal cortico-cortical connections, and the subcortical layers, which are less involved in horizontal connectivity, induces folding patterns similar to those in simulations of polymicrogyric brains (Budday et al., 2014a).

## 6. Discussion

Neurodevelopment involves a highly orchestrated sequence of events, which is tightly regulated by the complex interplay of various cell types both in space and time. The objective of this review was to summarize the timeline of human brain development and to correlate events on the cellular level to pattern selection and surface morphogenesis on the organ level. To bridge these biological scales, we have adopted a popular physical model for brain folding: the mechanism of differential growth.

Neurodevelopment begins with cell division and cell migration, which ultimately result in the formation of the characteristic six-layered cortex. Throughout this process, intermediate progenitor cells tend to fill the cortical layers radially, while radial glial cells tend to expand the cortex tangentially. From a mechanical point of view, unconstrained radial growth causes a thickening of the cortex, whereas constrained tangential expansion generates compressive stress, which may eventually induce cortical folding. When neuronal migration is mostly completed, cortical neurons begin to form connections with other neurons. Neuronal connectivity involves the growth of neuronal dendrites and axons, the generation and expansion of astrocytes, oligodendrocytes and microglial cells, the formation of synapses, and the development of the vasculature system. These phenomena collectively result in an excessive tangential expansion of the outer cortex, an increase in cortical stress, and a folding of the cortical layer. Postnatally, synaptogenesis, synaptic pruning, and myelination mediate further cortical and subcortical growth, alter the cortical stress state, and reshape the cerebral cortex. Disruption of any of these events can result in severe cortical malformations, which are associated with neurological disorders including developmental delay and epilepsy. Although gyrification occurs in synchrony with neuronal connectivity–after all neurons have reached their final position in the developing cortex–early neuronal migration has a significant influence on cortical folding. Neuronal migration sets the stage for the folding process; disturbed initial conditions trigger disrupted neuronal connectivity and cortical malformations.

Lissencephaly, for instance, originates from disrupted early migration, although its classical hallmark–the absence of folds–does not make an appearance until the neuronal connectivity initiates folding. The absence of folding in the lissencephalic brain can be explained by a fairly simple physics-based model for growth-induced mechanical instabilities: the compressive forces induced by neuronal connectivity are distributed over an extensively thickened cortex; the resulting tangential stress within the cortex, the force divided by the thickness, is therefore significantly smaller than in healthy brains and fails to initiate folding. Even if the connectivity process itself is entirely normal, the smooth cortex precludes the formation of short association fibers and normal functioning of the brain. Polymicrogyria, in contrast, is a true organization disorder. At the end of neural migration, the cortex exhibits regular initial conditions, but the connectivity process itself is disturbed. Increased cell death and damage of deeper cortical layers result in a cortex with small superficial folds. Although the origin of this pathology is entirely different from lissencephaly, the same physics-based model can explain its malformations: a locally thinned cortex and reduced tangential expansion in the deeper layers reduce the critical wavelength, and induce an increased number of small folds.

Advances in the physical modeling and computational simulation of living systems can give insights into the underlying mechanisms of cortical folding that complement our current knowledge of neurodevelopment. They allow us to bridge cellular phenomena and whole organ form and function. Naturally, computational models of brain development have to simplify the complex biological problem. The challenge is to capture the critical processes without oversimplifying the biological system. In this review, we have shown that a fairly simple physical model can predict how the mammalian cortex evolves under physiological and pathological conditions. This model explains cortical malformations as emergent properties of cellular disruptions, either during early cellular division and migration or during later cellular organization and connectivity.

## 7. Conclusion

Neurodevelopment is a complex, dynamic process that involves several contributing genetic, environmental, biochemical, and physical factors; it is unlikely to be deciphered by a single discipline alone. Researchers of various fields are beginning to come together to elucidate normal and abnormal brain development through close multidisciplinary collaborations. A traditional biological approach, for example, can identify cells and genes that regulate malformations of cortical development; yet, it fails to substantiate the classical hallmark of the disease, the malformed cortex. A physical approach cannot explain the genetic origin of cortical malformations; yet, it can explain growth-induced instabilities, quantify cortical stress, identify the critical stress necessary to induce folding, rationalize emergent folding patterns, and predict gyral wavelengths and gyrification indices. If calibrated and validated appropriately, physics-based models allow us to bridge the scales–from phenomena on the cellular level toward form and function on the organ level–and make quantitative predictions. Recent advances in neuroimaging would allow us to create high precision, personalized models for cortical folding. Combining biology and physics can help us advance our understanding of human neurodevelopment, identify early markers of cortical malformations, and, ultimately, improve treatment of developmental disorders including epilepsy, autism spectrum disorders, and schizophrenia.

## Author contributions

SB has performed the simulations, designed all figures, and written the manuscript. PS has discussed the outline and overseen the writing process. EK has implemented the initial computational method, discussed the outline, and mentored the writing process.

### Conflict of interest statement

The authors declare that the research was conducted in the absence of any commercial or financial relationships that could be construed as a potential conflict of interest.
